# Radiotherapy in Current Neuro-Oncology: There Is Still Much to Reveal

**DOI:** 10.3390/life11121412

**Published:** 2021-12-16

**Authors:** Gianluca Ferini, Giuseppe Emmanuele Umana

**Affiliations:** 1 REM Radioterapia srl, 95029 Catania, Italy; gianluca.ferini@grupposamed.com; 2 Department of Neurosurgery, Trauma Center, Gamma Knife Center, Cannizzaro Hospital, 95126 Catania, Italy

Radiation therapy (RT) has a pivotal role in the treatment of Central Nervous System (CNS) neoplasms and is routinely employed for both benign and malignant lesions [1]. Depending on the intent (i.e., curative or palliative), RT can be used alone or combined with surgery and systemic therapies [2]. Given CNS peculiarity, tumors at this site commonly have a dramatic onset with severe neurological impacts. When the clinical situation is particularly serious, prompt therapeutic action is required, especially debulking/decompressive surgical procedures [3]. However, some CNS tumors—especially the benign ones (i.e., meningiomas)—may be asymptomatic, often with an incidental diagnosis [4]. Classically, both primary and secondary malignant CNS tumors had a poor prognosis, and radiotherapy was mainly devoted to preserving the quality of life while avoiding iatrogenic injury. Indeed, nervous tissues have limited regenerative capability in response to radiation damage compared to other tissues (i.e., mucous membranes and skin) [5,6]; this is of particular concern among radiation oncologists, who must ponder every day the need for an escalated dose, such as that used in stereotactic ablative treatments, with the risk of serious adverse events (i.e., radionecrosis) [7]. In recent decades, this preliminary assessment has become particularly pervasive among insiders, since the recent advances in systemic therapy have prolonged the survival of cancer patients, making the scenario of controlled or controllable intracranial disease more common in radiotherapy departments [8]. Such a key point is of fundamental importance not only in the cerebral region but also in other neuroaxis-related sites; for example, any damage to the vertebral column could be life-altering. The risk for vertebral compression fracture is especially feared when treating bone metastases to the vertebral body with high radiation doses [9].

The effort to improve the RT therapeutic index is currently supported by new advancements in treatment delivery equipment and imaging techniques; the former is mainly directed at maximizing the sparing of healthy off-target tissues, while the latter are devoted to precisely defining the target or detecting tumor recurrences early, distinguishing them from post-radiotherapy changes [10]. Some imaging is also useful for the functional evaluation of irradiated brain tissue change [11]. This finding could serve to institute appropriate medical therapy before symptomatic progression in neurodegenerative clinical conditions.

GammaKnife (GK) and CyberKnife (CK) are two different radiotherapy medical devices that marked a turning point in the history of stereotactic brain radiosurgery, formerly exercised by classic monoisocentric Linear Accelerator (LINAC). While the first is exclusively devoted to the treatment of brain targets [12], the second is also used for treating extracranial tumors [13]. In recent years, a LINAC-based RT technique—namely Volumetric Modulated Arc Therapy (VMAT), which was typically employed for limiting radiation-related adverse events in extracranial sites [14,15]—has been enhanced with advanced Treatment Planning Systems (TPS) to plan a simultaneous radiation dose delivery to multiple brain metastases with maximal sparing of healthy tissue, similarly to what is achievable with GK [8]. Regarding the therapeutic index, these two stereotactic techniques constantly confront dosimetric challenges, and no clinical study has yet confirmed a clear benefit of the one over the other [16]. CK and GK are irreplaceably useful for RT treatments of extremely challenging targets wrapped around critical structures [17]. Finally, each RT technique has pros and cons, such as the dose delivery time. Indeed, the radiation oncologist must consider that a prolonged delivery time could negatively affect the patient’s compliance to treatment and, ultimately, clinical outcome. On the other hand, such a consideration introduces another revolutionary concept: an extremely shortened dose delivery time thanks to ultra-high dose rates could produce a convenient differential effect between tumor and healthy tissues. This idea is the basis of FLASH radiotherapy, whose preclinical results are already interesting and support further radiobiological investigations [18].

Proton therapy and, more broadly, adrotherapy are a milestone in precision RT history. By exploiting a peculiar dose absorption (Bragg peak), they allow us to raise radiation doses almost up to the target edge, with a steep dose fall-off beyond it [19]. To further enhance its potential, proton therapy could be adopted for unconventional use: an almost homogeneous high dose can be delivered deep into the target through a physical grid, whose submillimetric holes would permit sparing of the overlying healthy brain tissue between micron-sized beams [20]. Such a microbeam array fits into the broader framework of spatially fractionated radiotherapy (SFRT), whose biological mechanisms are currently generating great interest among scientists [21].

Considering that an increasing amount of new drugs is being launched, especially immuno- and molecular targeted therapies, neuro-oncologists need to be familiar with their possible interactions with concomitant RT, both in terms of improved clinical outcome and adverse events [22,23]. Even conceptually, new therapies, such as Tumor Treating Fields (TTFields), could be effectively integrated with RT for the treatment of highly aggressive brain tumors (e.g., glioblastoma) [24,25,26,27,28].

Ultimately, it is remarkable how a “drug” (ionizing radiation), whose debut in clinical practice is more than a century old, is still at the forefront in cancer research, including neuro-oncology. This means that RT could still have much more to offer. This special issue could be a good place for some of these interesting “revelations”.
